# Decrease of fear avoidance beliefs following person-centered progressive resistance exercise contributes to reduced pain disability in women with fibromyalgia: secondary exploratory analyses from a randomized controlled trial

**DOI:** 10.1186/s13075-016-1007-0

**Published:** 2016-05-21

**Authors:** Annie Palstam, Anette Larsson, Monika Löfgren, Malin Ernberg, Jan Bjersing, Indre Bileviciute-Ljungar, Björn Gerdle, Eva Kosek, Kaisa Mannerkorpi

**Affiliations:** Institute of neuroscience and physiology, Sahlgrenska Academy, University of Gothenburg, Gothenburg, Sweden; University of Gothenburg Centre for Person Centered Care (GPCC), Gothenburg, Sweden; Dep of Clinical Sciences, Karolinska Institutet and Dep of Rehabilitation Medicine, Danderyd Hospital, Stockholm, Sweden; Department of Dental Medicine, Karolinska Institutet, and Scandinavian Center for Orofacial Neurosciences (SCON), Huddinge, SE-141 04 Sweden; Sahlgrenska University Hospital, Rheumatology, Göteborg, Sweden; Department of Medical and Health Sciences, Faculty of Medicine and Health Sciences, Linköping University, Pain and Rehabilitation Centre, Linköping, Sweden; Department of Medical and Health Sciences, Linköping University, Linköping, Sweden; Department of Clinical Neuroscience, Karolinska Institutet and Stockholm Spine Center, Stockholm, Sweden

**Keywords:** Fibromyalgia, Pain, Disability, Resistance exercise, Person-centered

## Abstract

**Background:**

Fibromyalgia (FM) is characterized by persistent widespread pain, increased pain sensitivity and tenderness. Women with FM also report disability, in terms of negative consequences on activities of daily living. Our recent randomized controlled trial (RCT) is the first study of resistance exercise to show positive effects on pain disability. The resistance exercise program of our RCT emphasized active involvement of participants in planning and progression of the exercise, using the principles of person-centeredness, to support each participant’s ability to manage the exercise and the progress of it. The aim of this sub-study was to investigate explanatory factors for reduced pain disability in women with FM participating in a 15-week person-centered progressive resistance exercise program.

**Methods:**

A total of 67 women with FM were included in this sub-study of an RCT examining the effects of person-centered progressive resistance exercise performed twice a week for 15 weeks. Tests of physical capacity and health-related questionnaires were assessed at baseline and after the intervention period. Multivariable stepwise regression was used to analyze explanatory factors for improvements in pain disability.

**Results:**

Reduced pain disability was explained by higher pain disability at baseline together with decreased fear avoidance beliefs about physical activity (*R*^2^ = 28, *p* = 0.005). The improvements in the disability domains of recreation and social activity were explained by decreased fear avoidance beliefs about physical activity together with higher baseline values of each disability domain respectively (*R*^2^ = 32, *p* = 0.025 and *R*^2^ = 30, *p* = 0.017). The improvement in occupational disability was explained by higher baseline values of occupational disability (*R*^2^ = 19, *p* = 0.001).

**Conclusion:**

The person-centered resistance exercise intervention, based on principles of self-efficacy, had a positive effect on recreational, social and occupational disability. The reduced pain disability seemed to be mediated by decreased fear avoidance beliefs. Age, symptom duration, pain intensity, and muscle strength at baseline had no explanatory value for reduced pain disability, indicating that the person-centered resistance exercise program has the potential to work for anyone with FM who has interest in physical exercise.

The trial was registered on October 21, 2010 with ClinicalTrials.gov identification number: NCT01226784.

## Background

Disability is a complex concept that includes impairment, activity limitation, and participation restriction, reflecting the bio-psychosocial interaction between the person and the context in which he or she lives [1]. Musculoskeletal disorders are one of the most common reasons for work disability and sick leave in Sweden [2, 3] and hence, entail large costs for the individual and for society [4–6]. Fibromyalgia (FM) affects approximately 1–3 % of the general population, is more prevalent in older age [7, 8] and is six times more common in women than in men [9]. Women with FM are challenged by chronic pain, fatigue, psychological distress [10], and impaired physical capacity [11–14], and report consequences on their activities of daily life and difficulties in fulfilling their life roles [15, 16], including their ability to work [14, 17–20]. Reduced physical capacity and pain in FM appear to contribute to participation restriction and disability [14, 21]. Further, the physical demands at work constitute substantial problems for persons with musculoskeletal pain to manage their work and entail increased risks of work disability and sick leave [22, 23]. Also, women with FM describe their impaired physical capacity as being a hindrance for managing physical work demands [24].

Women with FM have been shown to be less physically active than healthy women, especially in moderate to high intensity physical activity [25]. The relationship between pain and muscle weakness in FM is not entirely understood but may to some degree be explained by exercise-induced pain [26]. Pain is an obstacle for patients to engage in exercise of higher intensity [27] and could also raise fear avoidance beliefs about physical activity that may lead to a vicious circle of inactivity and disability [28].

Individually tailored physical exercise is recommended for improving physical capacity and participation in daily life activities in FM [29–33]. Previous studies of resistance exercise in women with FM have observed improved muscle strength [34–38], and reduced pain intensity following resistance exercise [37–40]. Our recently published randomized controlled trial (RCT) is the first study of resistance exercise in FM to also show positive effects on pain disability assessed as the total score of the pain disability index (PDI) [37]. However, changes in the separate subscales assessing different domains of pain disability were not investigated. The resistance exercise program focused on the active involvement of each participant in planning and progressing the exercise program, using the principles of person-centeredness. This theory emphasizes the partnership between participant and physiotherapist and shared decision-making based upon the participant’s descriptions of their wishes, needs, and resources, and this approach is suggested to support each participant’s ability to manage the exercise and the progress of it, and subsequently to enhance the patient’s ability to manage their health problems [41].

The factors interacting with reduced pain disability in women with FM engaging in resistance exercise have to the best of our knowledge not previously been investigated. The aim of this sub-study was to investigate explanatory factors for improvements in aspects of pain disability in women with FM, participating in a 15-week person-centered progressive resistance exercise program.

## Methods

### Study design

This was a sub-study of an assessor-blinded randomized controlled multi-center trial, which examined the effects of progressive resistance group exercise compared with an active control group. The trial was registered on 21 October 2010 with ClinicalTrials.gov identification number: NCT01226784 [37]. The study was approved by the regional ethics committee in Stockholm (2010/1121-31/3). Written and oral information was given to all participants and written consent was obtained from all participants.

### Participants

The recruitment started in 2010 and data collection was completed at all sites (Gothenburg, Stockholm, and Linköping) in 2013. Inclusion and exclusion criteria have been described in detail previously [37]. In short, the inclusion criteria were women aged 20–65 years, meeting the American College of Rheumatology (ACR) 1990 classification criteria for FM and the exclusion criteria were other severe somatic or psychiatric disorders, participation in a rehabilitation program within the past year, or inability to understand Swedish. Women with FM were recruited to the multi-center experimental study by newspaper advertisement in the local newspapers of three cities in Sweden (Gothenburg, Stockholm, and Linköping) [37]. One-hundred and thirty women with FM were included in the multicenter experimental study, of whom 67 were randomized to the resistance exercise group. The process of recruitment and randomization has been described in detail in the previous publication [37]. All participants were invited to a post-treatment examination after the 15-week intervention period and 84 % (n = 56) of the women in the resistance exercise group completed the examination. Five participants in the resistance exercise group reported adverse effects and chose to discontinue the intervention due to increased pain. Out of these, two participants completed the post-treatment examinations [37].

### Resistance exercise intervention

The person-centered progressive resistance exercise intervention was performed twice a week for 15 weeks at physiotherapy premises and at a local gym, in small groups and supervised by experienced physiotherapists. The exercise sessions started with a 10-minute warm up followed by 50 minutes of resistance exercises focusing on large muscle groups in all four extremities and trunk. The resistance exercise was initiated at low loads corresponding to 40 % of one repetition maximum (RM) and progressed up to 80 % of one RM during the 15 weeks. Possibilities for progressions of loads were evaluated every 3–4 weeks. Forty-two participants (62.7 %) reached exercise loads of 80 % of 1 RM while seven participants (10.4 %) reached exercise loads of 60 % of 1 RM. The median attendance rate at the exercise sessions was 71 % (range 0–100 %). A more detailed description of the procedure of the resistance exercise and the adjustment of loads has been described previously [37].

### Assessments

Outcomes were assessed at baseline and immediately after the 15-week intervention period. All participants were invited to post-treatment examinations according to an intention-to-treat design. Examinations included self-reported questionnaires, performance-based tests of muscle strength, and assessment of current pain intensity. Background data were gathered using a standardized interview. Examinations were conducted at physiotherapy premises by physiotherapists who were blinded to group allocation. Baseline and post-treatment examinations were performed by the same physiotherapists.

### Measures included in analyses

The dependent variables followed by the independent variables included in the analyses of explanatory factors for improvement in the pain and disability are subsequently presented.

### Dependent variable

#### Pain disability

The Pain Disability Index PDI was used to assess the impact that pain has on the ability of a person to participate in essential life activities on a scale from 0–70. A higher score indicates greater disability. The PDI includes seven subscales: (1) family and home responsibilities, covering activities related to home and family; (2) recreation, covering hobbies, sports and other leisure time activities; (3) social activity, covering participation with friends and acquaintances other than family members; (4) occupation, covering activities partly or directly related to working, including housework or volunteering; (5) sexual behavior, covering frequency and quality of sex life; (6) self care, covering personal maintenance and independent daily living (bathing, dressing, etc.); and (7) life-support activity, covering basic life-supporting behavior (eating, sleeping, breathing, etc.) [42, 43]. The PDI has shown satisfactory test-retest reliability and is valid for patients with chronic pain [42–44].

### Selection of independent variables

Variables that were included as potential predictors or explanatory factors for change in the dependent outcome variables were as follows. Age and symptom duration were selected because older age could be anticipated to influence the effect of the resistance exercise and a longer symptom duration often means a prolonged period of decrease in the level of physical activity, which could potentially influence the level of disability.

#### Pain intensity

Pain intensity correlates with the degree of disability in FM [45], and it is reasonable to assume that change in pain intensity would influence the degree of disability. Current pain intensity was measured by a visual analog scale (VAS). A 100-mm plastic VAS with a moveable cursor along a line and anchors at the extremes only (“no pain at all” to “worst imaginable pain”), was used. The participant was asked to assess her current pain ranging from “no pain at all” to “worst imaginable pain”. The VAS has been reported to be a useful measure of pain intensity in most settings [46].

#### Muscle strength

Muscle strength is reduced by 20–40 % in FM, and previous studies have shown a correlation between muscle strength and disability in FM [45]. It was hypothesized that improved muscle strength might improve disability. Isometric knee-extension force (N) was measured with a dynamometer (Steve Strong®: Stig Starke HBI, Göteborg, Sweden) using a standard protocol. The participant was in a fixed seated position with back support, knee and hip in 90° of flexion, and legs hanging freely. A non-elastic strap was placed around the ankle and attached to a pressure transducer with an amplifier. The subjects were instructed and verbally encouraged to pull the ankle strap with maximal force for 5 seconds. Three trials were performed for each test with a 1-minute rest between each trial. The best performance out of three trials was recorded.

Hand-grip force (N) was registered bilaterally using Grippit® (AB Detektor, Göteborg, Sweden). The mean force over 10 seconds was recorded [47]. Two trials were performed for each test with a 1-minute rest between each trial. The best performance out of two trials was recorded.

#### Physical activity

Exercise is considered to be an important part of rehabilitation in FM, and it was of interest to study if the amount of physical activity was related to disability. The leisure time physical activity instrument (LTPAI) was used to assess the amount of physical activity performed during a typical week, reported in hours. The total score is the sum of hours spent on the activities [48].

#### Fear avoidance beliefs about physical activity

The theoretical construct of fear avoidance is an interesting concept in exercise interventions, as fear of physical activity was hypothesized to change during the intervention as the participants confronted with their pain at each session, which is the opposite to avoiding pain. The fear avoidance beliefs questionnaire, physical subscale (FABQ_physical_) was used to assess the extent to which fear and avoidance affect physical beliefs (four items, 0–24). A higher score represents more fear avoidance beliefs [49].

### Statistics

Data were computerized and analyzed using the Statistical Package Software for the Social Sciences (SPSS version 22.0, Chicago, IL, USA). Descriptive data are presented as mean, standard deviation (SD), median (min; max) for continuous variables or the number (*n*) and percentage (%) for categorical variables. All significance tests were two-sided and conducted at the 5 % significance level. Outcomes were analyzed according to the intention-to-treat design, implying that all participants were invited to post-treatment examination, whether they had participated in the intervention or not. Only measured values were included in analyses of changes over time, implying that cases missing were not included in the analysis. The Wilcoxon signed rank test was used to analyze changes from baseline to post-treatment examinations in the PDI total score and the PDI domains within the resistance exercise group.

Correlation between the dependent variables of change in pain disability and baseline measures and measures of change in the independent variables was tested by calculating the Spearman correlation coefficient. Variables with a *p* value <0.2 on analysis of correlation were included in further analyses using multiple linear forward stepwise regression analysis. The assumptions of normality were confirmed by checking the residual scatter plots and histograms of each variable respectively. Linear forward stepwise multiple regression analysis was performed to analyze explanatory factors for improvements in and pain disability.

## Results

### Results reported elsewhere and used in this study

#### Characteristics of participants

The mean age of the participants was 51 (SD 9.1) years (n = 67). Their mean symptom duration was 11 (SD 8.5) years (n = 67). A total of 38 (57 %) women worked to some extent and 2 (3 %) were full-time students, while 22 (34 %) did not work due to sick leave and 5 (7 %) did not work due to unemployment.

#### Pain disability

Mean pain disability at baseline was 35.3 (SD 12.2) (n = 67). Participants rated significantly reduced pain disability (*p* = 0.006) at post-treatment examination compared to baseline (Δ –3.8, SD 10.6) (n = 56) [37].

#### Pain intensity

The mean pain intensity at baseline was 49 (SD 23.9) mm (n = 67). Pain intensity was significantly reduced (*p* = 0.002) from baseline to post-treatment examination (Δ –5.7, SD 15.0) (n = 56) [37].

#### Muscle strength

The mean isometric knee-extension force at baseline was 330 (SD 109.4) N (n = 67). Isometric knee-extension force improved significantly (*p* = 0.002) from baseline to post-treatment examination (Δ 30.4, SD 71.9) (n = 56) [37]. The mean hand-grip force at baseline was 162 (SD 68.7) N (n = 67). Hand-grip force improved significantly (*p* < 0.001) from baseline to post-treatment examination (Δ 20.1, SD 36.1) (n = 52) [37].

#### Physical activity

The mean amount of physical activity at baseline was 5.6 (SD 4.8) hours (n = 67). The amount of physical activity increased significantly (*p* < 0.001) from baseline to post-treatment examination (Δ 2.3, SD 4.8) (n = 56) [37].

#### Fear avoidance beliefs

The mean fear avoidance beliefs about physical activity were 9.7 (SD 6.1) (n = 67). At the individual level, 23 % (15 out of 67) of the women displayed elevated fear avoidance beliefs corresponding to a fear avoidance score of >14 [50] at baseline, and 17 % (9 out of 54) displayed elevated fear avoidance beliefs at the post-treatment examination. Fear avoidance beliefs about physical activity did not improve significantly (*p* = 0.36) from baseline to post-treatment examination (Δ –0.8, SD 7.0) (n = 54) [37].

### Within-group analysis of change in pain disability

The PDI total score and the domains of recreation, social activity and occupation improved significantly from baseline to post-treatment examinations (Table 1).

**Table 1:** Analysis of change in pain disability from baseline to post-treatment examinations

Pain disability index (PDI)	Baseline (n = 67)	Post-test–baseline (n = 56)	Statistics
	Mean (SD) Median (min; max)	Δ Mean (SD) Median (min; max)	*P* value
PDI total (0–70)	35.25 (12.23) 36 (8; 69)	−3.84 (10.56) −4.5 (−29; 23)	**0.006**
PDI family and home (0–10)	5.39 (2.07) 6 (1; 10)	−0.38 (2.32) 0 (−6; 5)	0.24
PDI recreation (0–10)	6.55 (2.13) 7 (1; 10)	−0.91 (2.11) −1 (−6; 4)	**0.002**
PDI social activity (0–10)	5.21 (2.41) 5 (1; 10)	−0.70 (2.31) −0.5 (−5; 5)	**0.034**
PDI occupation (0–10)	5.94 (2.70) 7 (1; 10)	−0.59 (2.35) −0.5 (−6; 7)	**0.031**
PDI sex life (0–10)	5.71 (2.52) 6 (1; 10)	−0.46 (2.20) 0 (−8; 5)	0.156
PDI self care (0–10)	3.36 (2.25) 3 (1; 9)	−0.45 (1.88) 0 (−6; 5)	0.071
PDI life-support activity (0–10)	3.16 (2.52) 2 (1; 10)	−0.59 (2.92) 0 (−8; 6)	0.10

Missing values: PDI sex life (n = 2). Δ refers to change between post-test and baseline values. *P* values in bold type were significant

### Explanatory factors for reduced pain disability

The results of the correlation analyses are presented in Table 2. Variables that were found to correlate with pain disability with a *p* value <0.2 were included in the multiple linear regression analyses of explanatory factors for change in pain disability. The results of the regression analyses of explanatory factors are presented in Table 3.

**Table 2:** Analyses of correlations between the changes in pain disability and the independent variables (n = 56)

Independent variables	Δ PDI total	Δ PDI recreation	Δ PDI social	Δ PDI occupation
	*r*_s_ (*p* value)	r_s_ (*p* value)	*r*_s_ (*p* value)	*r*_s_ (*p* value)
Age (years)	0.16 (0.23)	0.05 (0.74)	−0.02 (0.88)	0.17 (0.21)
Symptom duration (years)	0.03 (0.81)	−0.12 (0.37)	0.12 (0.37)	0.17 (0.22)
Pain intensity (0–100)	**−0.26 (0.054)** ^**a**^	**−0.19 (0.17)** ^**a**^	−0.04 (0.75)	**−0.06 (0.67)** ^**a**^
PDI total (0–70)	**−0.34 (0.01)**	**−0.25 (0.066)** ^**a**^	**−0.25 (0.067)** ^**a**^	**−0.18 (0.19)** ^**a**^
PDI recreation (0–10)	-	**−0.30 (0.027)**	-	-
PDI social (0–10)	-	-	**−0.45 (<0.001)**	-
PDI occupation (0–10)	-	-	-	**−0.37 (0.005)**
LTPAI (hours)	**0.19 (0.17)** ^**a**^	0.09 (0.54)	0.04 (0.75)	−0.01 (0.99)
Knee-extension force (N)	−0.13 (0.32)	0.04 (0.75)	−0.07 (0.61)	−0.15 (0.28)
Hand-grip force (N)	−0.05 (0.70)	0.11 (0.43)	−0.01 (0.99)	−0.061 (0.65)
FABQ_physical_ (0–24)	0.01 (0.93)	0.04 (0.75)	**−0.18 (0.19)** ^**a**^	0.01 (0.94)
Δ Pain intensity (0–100)	**0.36 (0.008)**	**0.35 (0.008)**	**0.18 (0.19)** ^**a**^	0.15 (0.26)
Δ LTPAI (hours)	**−0.28 (0.037)**	−0.11 (0.43)	**−0.27 (0.049)**	−0.05 (0.72)
Δ Knee-extension force (N)	**−0.32 (0.019)**	−0.16 (0.25)	−0.12 (0.39)	**−0.23 (0.09)**
Δ Hand-grip force (N)	**−0.23 (0.10)** ^**a**^	−0.14 (0.33)	**−0.25 (0.08)**	**−0.32 (0.023)**
Δ FABQ_physical_ (0–24)	**0.36 (0.007)**	**0.44 (0.001)**	**0.34 (0.012)**	0.17 (0.23)

Missing values: fear avoidance beliefs questionnaire (FABQ)_physical_ (n = 1), Δ FABQ_physical_ (n = 2), Δ pain disability index (PDI) social (n = 2), Δ PDI occupation (n = 2), Δ Hand-grip force (n = 4): Δ refers to change between post-test and baseline values. *LTPAI*, leisure time physical activity instrument. Results for variables with a *p* value <0.05 on analysis of correlation were considered significant and are marked in bold type. ^a^Variables with a *p* value <0.2 on analysis of correlation, which were then included in multiple stepwise regression analysis

**Table 3:** Explanatory models for improvement in pain disability (n = 56)

Explanatory model for change in pain disability	*R* square	Unstandardized coefficients	*P* value
		B (Std error)	
1 Δ FABQ_physical_	0.28	0.52 (0.18)	0.006
2 PDI total		−0.32 (0.11)	0.005
Explanatory model for change in PDI recreation	*R* square	Unstandardized coefficients	*P* value
		B (Std error)	
1 Δ FABQ_physical_	0.32	0.13 (0.04)	0.001
2 PDI recreation		−0.28 (0.12)	0.025
Explanatory model for change in PDI social activity	*R* square	Unstandardized coefficients	*P* value
		B (Std error)	
1 PDI social	0.30	−0.42 (0.11)	<0.001
2 Δ FABQ_physical_		0.10 (0.04)	0.017
Explanatory model for change in PDI occupation	*R* square	Unstandardized coefficients	*P* value
		B (Std error)	
1 PDI occupation	0.19	−0.38 (0.11)	0.001

Missing values: regression model for change in pain disability (n = 2), regression model for change in pain disability index (PDI) recreation (n = 2), regression model for change in PDI social activity (n = 2), regression model for change in PDI occupation (n = 2). Δ refers to change between post-test and baseline values. *FABQ*_*physical*_ fear avoidance beliefs questionnaire, physical subscale, *Std error* standard error

### Pain disability (PDI total)

Higher pain disability at baseline together with improvement in fear avoidance beliefs about physical activity were found to partly explain improvement in pain disability. This model explained 28 % of the improvement in pain disability (*p* = 0.005) (Table 3).

### PDI recreation

Improvement in fear avoidance beliefs about physical activity together with higher baseline values of PDI recreation were found to partly explain improvement in PDI recreation. This model explained 32 % of the improvement in PDI recreation (*p* < 0.025) (Table 3).

### PDI social activity

Higher baseline values of PDI social activity together with improvement in fear avoidance beliefs about physical activity were found to partly explain improvement in PDI social activity. This model explained 30 % of the improvement in PDI social activity (*p* < 0.017) (Table 3).

### PDI occupation

Higher baseline values of PDI occupation were found to partly predict the improvement in PDI occupation. This model predicted 19 % of the improvement in PDI occupation (*p* = 0.001) (Table 3).

## Discussion

The pain disability total score and three out of the total of seven domains of pain disability improved with resistance exercise, the improved domains being recreation, social activity, and occupation. These domains are regarded as activities that require more physical capacity, which was reflected by the baseline scores of these domains being higher compared to the baseline scores of the domains of self-care and life support activities, which can be regarded as less physically demanding activities [43]. The less physically demanding activities did not improve with resistance exercise, implying that the women had sufficient capacity for performing these activities to start with, and therefore the performance was not influenced by resistance exercise. The mean score for pain disability at baseline was 35.3 (0–70), which is in line with recently published reference values for disability scores (PDI) in patients with chronic musculoskeletal pain [51]. A total of 54 % (n = 30) of the participants improved their pain disability by more than 12 % from baseline to post-treatment examinations, which corresponds to a clinically relevant improvement in pain disability [52].

Occupational disability improved significantly with resistance exercise, which is an important result for most women with FM who are lacking sufficient physical capacity to perform their work tasks and facing the risk of long-term work disability [53, 54]. This improvement could contribute to future return to work among the women currently on sick leave or decreased risk of future sick leave among the women currently working. However, return to work is a complex process and no significant changes in actual sick leave were observed.

The only variable that explained improvement in pain disability due to resistance exercise was decrease in fear avoidance beliefs about physical activity, besides higher baseline values of pain disability, which was expected. The results imply that fear avoidance beliefs are an important factor to take into consideration, and strategies to diminish fear while performing physical exercise need to be included in an exercise program. However, the models only explain 19–32 % of the improvement in pain disability, implying that other unknown factors also contribute to the change.

The primary reason for the graded progression of loads in the individualized resistance exercise program was to avoid exercise-induced pain. However, from a fear avoidance perspective, the progression seems to also have worked as a graded exposure to physical activity, which has previously been reported to reduce fear avoidance beliefs in patients with FM [55].

Fear avoidance beliefs about physical activity did not, however, improve significantly in the women participating in the resistance exercise program, as reported in our previous RCT [37]. One reason as to why the fear avoidance beliefs did not improve significantly with resistance exercise might be that the levels of fear avoidance were relatively low at baseline, mean 9.71, SD 6.08, on a scale ranging from 0–24 [49]. The cutoff score for elevated fear avoidance beliefs in physical activity have previously been defined as >14 (0–24) [50]. When comparing the levels of fear avoidance beliefs of the participants in our study at the individual level with this cutoff we found that the baseline scores of 23 % of the women in our study population corresponded to elevated fear avoidance beliefs, while the percentage was 17 % at post-treatment examinations.

The improvement in pain disability due to resistance exercise can be assumed to be related to improvement in physical capacity and the participants becoming increasingly motivated to exercise with heavy loads. This process was facilitated by the person-centered approach [41] of the intervention, based on principles of increasing self-efficacy [56]. To give the participant a sense of control in a given situation is an important component to enhance self-efficacy and this was achieved by the active involvement of the participant in adjusting and modifying the exercises and loads and in the progression of the resistance exercise [57]. The exercise was initiated at low loads during an extended time period, to allow for a positive experience of the exercise and a slow adaptation of the participants’ physical capacity to avoid exercise-induced pain. Performing the exercise program in small groups together with others sharing similar difficulties and to see them manage the exercise is another of the key components of self-efficacy enhancement [56], and hence, it is beneficial for persons with FM to exercise in groups. Also, encouragement and physiological feedback from physiotherapists supervising the groups is assumed to support self-efficacy to manage disabilities while exercising [56].

The fact that age, symptom duration, pain intensity, and muscle strength at baseline had no explanatory value for reduced pain disability indicates that the effects of this resistance exercise program did not depend on the characteristics of each individual participant, but rather that the intervention has the potential to be effective for anyone with FM, who is interested in exercise, which was the idea when assuming a person-centered approach to the intervention.

## Limitations

The recruitment procedure i.e., newspaper advertisement may have resulted in recruitment of participants who were motivated to exercise and this could bias the results. To minimize this risk the advert was designed to recruit participants to both interventions so none of the participants would know in advance which intervention was the active intervention or the control intervention.

## Conclusions

The person-centered resistance exercise intervention, based on principles of self-efficacy, had a positive effect on recreational, social, and occupational disability. The reduced pain disability appeared to be mediated by decrease in fear avoidance beliefs together with higher baseline scores for pain disability; however, these results are exploratory and need replication.

## Abbreviations

FABQ: fear avoidance beliefs questionnaire
FABQ_physical_: fear avoidance beliefs questionnaire, physical subscale
FM: fibromyalgia
LTPAI: leisure time physical activity instrument
PDI: pain disability index
RCT: randomized controlled trial
RM: repetition maximum
VAS: visual analog scale
